# Lower Beclin 1 downregulates HER2 expression to enhance tamoxifen sensitivity and predicts a favorable outcome for ER positive breast cancer

**DOI:** 10.18632/oncotarget.11044

**Published:** 2016-08-04

**Authors:** Yu Gu, Tianxiang Chen, Guangliang Li, Cong Xu, Zhenzhen Xu, Jing Zhang, Kuifeng He, Linyan Zheng, Zhonghai Guan, Xinyun Su, Jiang Cao, Lisong Teng

**Affiliations:** ^1^ Department of Radiation Oncology, Shanghai Cancer Hospital, Fudan University, Shanghai, China; ^2^ Department of Surgical Oncology, The 1st Affiliated Hospital, School of Medicine, Zhejiang University, Hangzhou, Zhejiang Province, China; ^3^ Department of Thoracic Surgery, Shanghai Chest Hospital, Shanghai Jiao Tong University, Shanghai, China; ^4^ Department of Medical Oncology, Zhejiang Cancer Hospital, Hangzhou, Zhejiang Province, China; ^5^ Clinical Research Center, The Second Affiliated Hospital of Zhejiang University School of Medicine, Hangzhou, Zhejiang Province, China

**Keywords:** breast cancer, tamoxifen resistance, prognosis, beclin 1, HER2

## Abstract

Tamoxifen(TAM) is one of the most effective endocrine treatment for estrogen receptor(ER)-positive breast cancer, however drug resistance greatly limits benefit of it. Our purpose is to uncover the role of Beclin 1 in tamoxifen resistance and prognosis of ER positive breast cancer. We established a tamoxifen resistant ER-positive breast cancer cell subline MCF-7R presenting with higher Beclin 1 and human epidermal growth factor receptor 2(HER2) levels than MCF-7. Silencing Beclin 1 decreased levels of HER2 and significantly promoted TAM sensitivity of MCF-7 and MCF-7R *in vitro*. Overexpression of HER2 could reverse TAM sensitivity, which was formerly increased in Beclin 1 downregulated cell. Beclin 1 level was not only positively correlated with level of HER2 but also negatively correlated with overall survival of ER-positive breast cancer patients. Using bioinformatic methods, Beclin 1 mRNA was found to be negatively correlated with overall survival in breast cancer patients receiving TAM treatment. This study indicated for the first time that lower HER2 expression by Beclin 1 downregulation contributes to alteration of tamoxifen sensitivity and low Beclin 1 predicts favorable outcome in ER-positive breast cancer.

## INTRODUCTION

Breast cancer is the most frequent and the second death cause of cancer among females [1] and approximately 70% human breast cancers are estrogen receptor(ER)-positive [2]. Endocrine drugs are still the key treatment for ER-positive breast cancer patients preoperatively, post-operatively, or during the metastatic disease stage. Endocrine drugs function by reducing estrogen levels or blocking ER signaling, which including selective estrogen receptor modulators (SERMs), selective estrogen receptor down-regulators (SERDs), and aromatase inhibitors (AIs) etc. Tamoxifen(TAM), as one of the SERMs, is the most frequently used endocrine drug which can competitively block ER to inhibit estrogen signaling and tumor growth [3]. A recent meta-analysis reported that 5 years of adjuvant TAM reduced 15-year risks of breast cancer recurrence and death from the data of 21,457 patients [4]. However, *de novo* and acquired resistance which occur in about 30% of the patients, seriously hinder the benefit of TAM [5, 6, 7].

The underlying mechanisms for TAM resistance are multifactorial and remain largely unknown. To date, a growing number of evidences have suggested that increased growth factor pathway, particularly EGFR/HER2 signaling, contributes to TAM resistance [8]. The direct interaction of ER and HER2 may contribute to the protection of HER2-overexpressed breast cancer cells from TAM-induced apoptosis [9]. Recent clinical studies suggest that *HER2* gene amplification and/or expression in patients with ER-positive breast cancer may be associated with TAM resistance [10–13]. Since TAM may activate a HER2-signalling pathway which may cause drug resistance, it is feasible that TAM combined with therapy against HER2-related pathway would be good for the patients with ER-positive breast cancer. [8, 14–20]

*Beclin 1* gene was firstly discovered by Levine et al. in 1998. It is located in human chromosome 17 q21 [21] and is the homolog of the yeast Atg6/Vps30 which is important in autophagy and vacuolar protein sorting [22, 23]. Especially, Beclin 1 is a key role in human pathogenesis especially in cancer [24, 25], however, expression and function of Beclin 1 in different cancers, even in different stages of the same cancer, was not consistent. For example, studies have shown that higher Beclin 1 expression suggested better overall survival in patients with non-Hodgkin's lymphoma, salivary gland carcinoma and gastric cancer [26–28]. While in patients with endometrial carcinoma, higher expression of Beclin 1 was associated with poorer prognosis [29]. Interestingly, Koukourakis et al. found that colon cancer patients with extremely higher or lower Beclin 1 expression level have worse prognosis than other patients [30]. Beclin 1 expression is also closely related with the effect of tumor treatments. With 5-fluorouracil treatment, colon cancer patients with higher expression of Beclin 1 had poorer overall survival [31]. A randomized controlled study in patients with nasopharyngeal carcinoma receiving radiation and chemotherapy found that higher expression of Beclin 1 associated with lower overall survival and progression-free survival [32].

The role of Beclin 1 in breast cancer exists different opinions. In ER positive HER2-negative breast cancer, Dong et al. found lower Beclin 1 expression indicated worse prognosis [33]. However, Gong et al. found that Beclin 1 was the key factor to maintain cancer stem cell activity and tumor development in breast cancer [34]. Recent studies found that Beclin 1 could down-regulate estrogenic signaling and growth response which lead to antiestrogen resistance. [35]. All above suggested that Beclin 1 might be related to treatment and prognosis of estrogen receptor-positive breast cancer. Despite of its well-known role in autophagy, additional membrane-trafficking function of Beclin 1 was also reported to be important in tumors. [36, 37]. Beclin-1 has been reported to interact with various of membrane receptors or their adaptors [35, 38, 39, 40].

Because of the importance of membrane growth factor receptor HER2 signaling in ER-positive breast cancer, we assessed relationship between Beclin 1 and HER2 and the role of Beclin 1 in ER-positive breast cancer. The data we report here reveal a novel role for Beclin 1 in the tamoxifen resistance and patients survival in ER-positive breast cancer.

## RESULTS

### TAM-resistant breast cancer cells (MCF-7R) exhibited increased levels of Beclin 1 and HER2 expression

**Figure 1:**
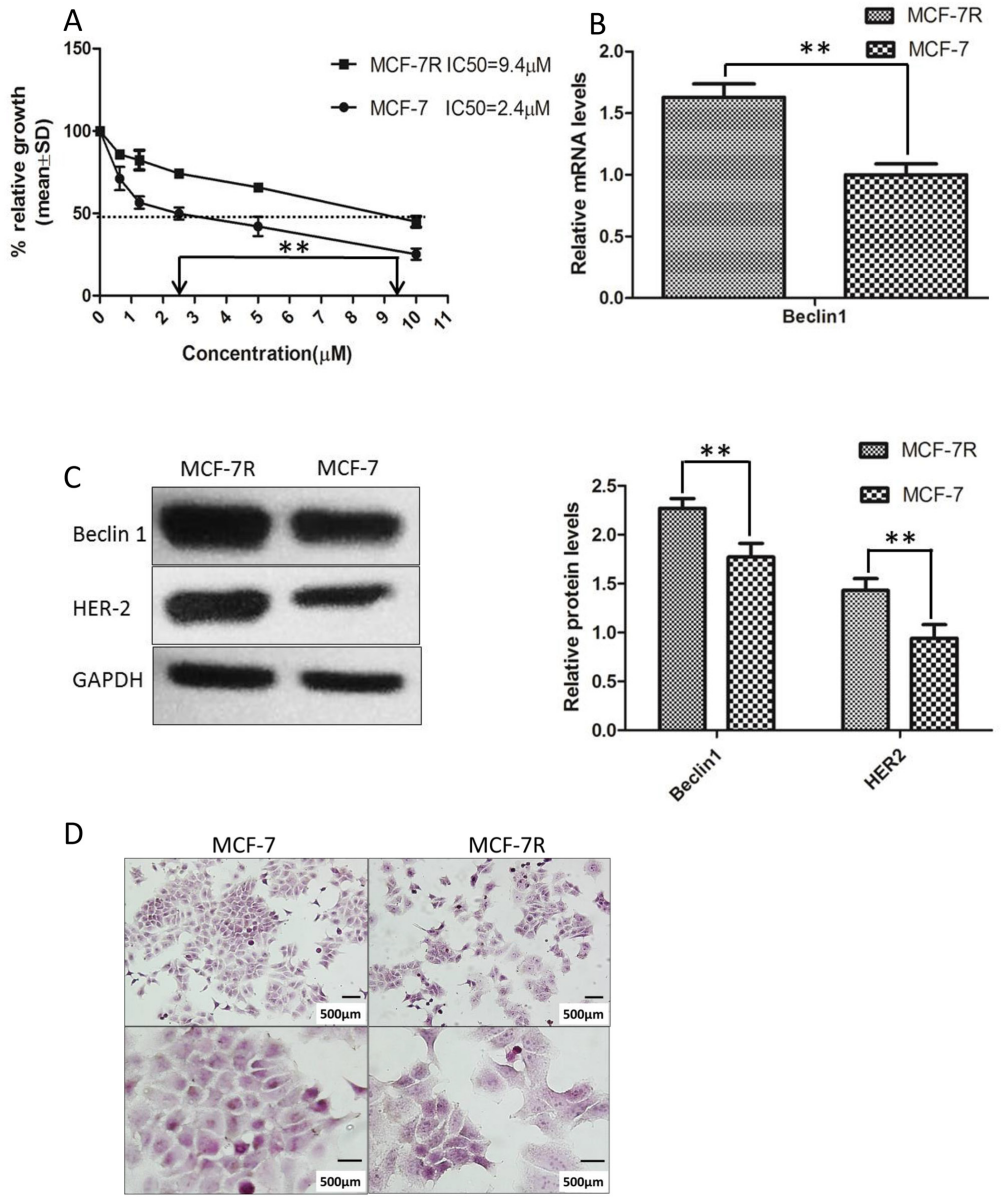
Tamoxifen-resistant MCF-7 cells (MCF-7R) was established and possessed increased levels of Beclin 1 and HER2 **A.** Tamoxifen (TAM) IC50 was examined by MTT analysis after incubation of the cells in the indicated concentrations of the drug (0.5–10 μM). The IC50 value for MCF-7 was 2.4 μM and 9.4 μM for MCF-7R. Each data point represent values from three independent experiments (n = 3). The dotted arrow represents where 50% inhibition of growth intercepts with the X axis and was used to estimate the IC50. The statistical significance was determined by the t test (**p<0.01). **B.** Representative mRNA expression of Beclin 1 in MCF-7R and MCF-7 cell.β-actin was used for normalization. **p < 0.01. **C.** Western blotting analysis of the protein levels of Beclin 1 and HER2 in MCF-7 cells and MCF-7/R cells (left). All experiments were repeated at least three times, and the quantitative results were analyzed by Image J software (right), and GAPDH was used as an endogenous control. **p < 0.01. **D.** Cell morphology of MCF-7 cells and MCF-7R cells. Scale bar= 500 μm.

Currently, TAM resistance remains a major challenge in breast cancer treatment [41]. To study the underlying molecular mechanism we established a ER-positive TAM resistant cell subline (MCF-7R) according to previous studies [42]. The 50% inhibitory concentration (IC50) values of TAM were then examined using MTT assay. Compared to parental MCF-7 cells, MCF-7R cells exhibited dramatically decreased sensitivity to TAM (IC50: 9.4μm versus 2.4μM, p<0.01) (Figure 1A). Moreover, we observed that the mRNA and protein levels of Beclin 1 in MCF-7R cells were increased compared to parental MCF-7 cells. Expression of Beclin 1 mRNA showed > 1.6 fold higher and Beclin 1 protein showed >1.3 fold higher in MCF-7R cells compared to MCF-7 cells (Figure 1B, 1C). Previous studies suggested that HER2 elevation was involved in the generation of acquired TAM resistance in breast cancer [43, 44]. Up-regulation of HER2 expression was also observed in MCF-7R cells in our study (Figure 1C). Meanwhile, cell morphology of MCF-7R was more polygonal and bigger size than MCF-7 (Figure 1D).

### Downregulating Beclin 1 expression by siRNA increased TAM sensitivity of breast cancer cells, which led to decreased proliferation, increased cell apoptosis, as well as lower migratory and invading capabilities

**Figure 2:**
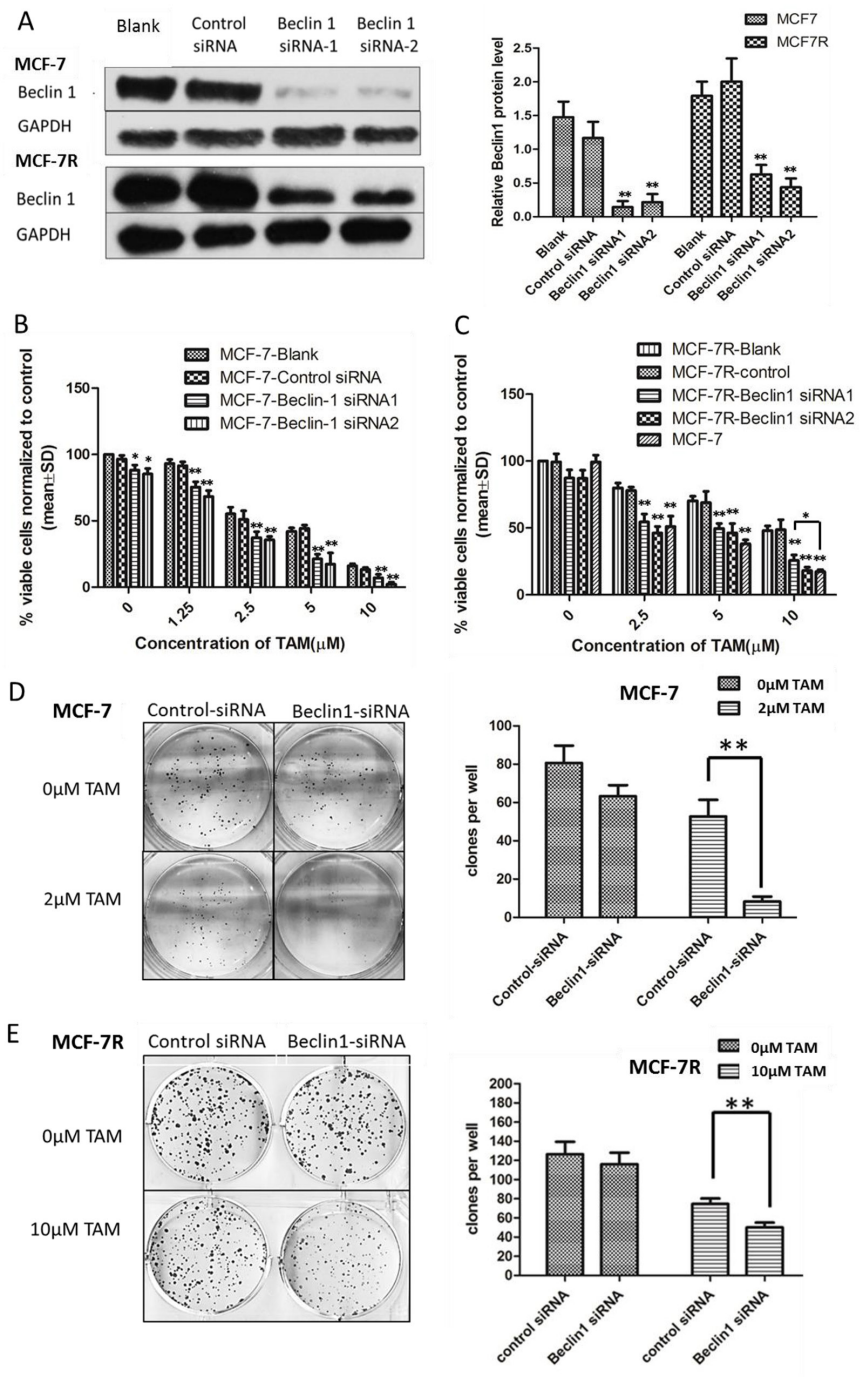

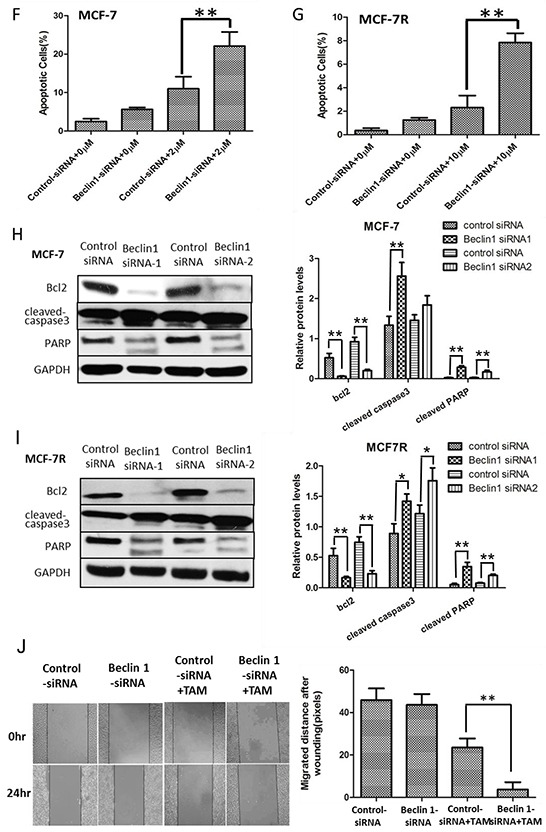

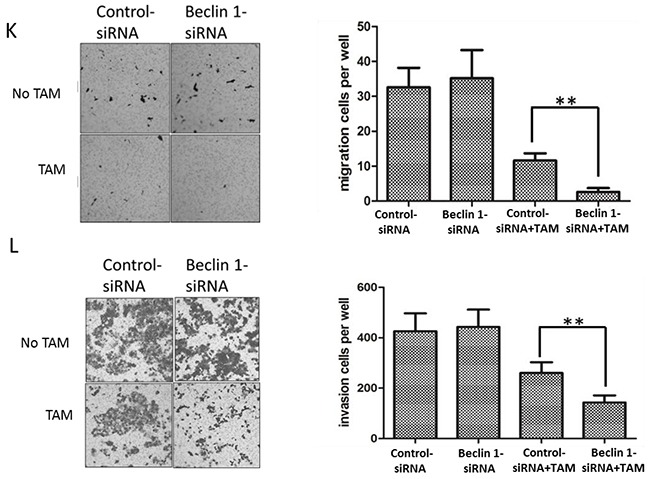
Beclin 1 downregulation led to increased tamoxifen sensitivity of ER-positive breast cancer cells including decelerated proliferation, enhanced cell apoptosis together with lower migration and invasion capability **A.** Western blot for Beclin 1 of MCF-7 cells and MCF-7R cells transfected with Beclin 1 siRNA, control siRNA or no siRNA (left). All experiments were repeated at least three times, and the quantitative results were analyzed by Image J software (right), and GAPDH was used as an endogenous control. **p < 0.01, one-way ANOVA. **B-C.** MTT assay of proliferation in MCF-7 and MCF-7R cells expressing either Beclin 1 siRNA or control siRNA in the presence of different concentrations of tamoxifen or not. *p<0.05,**p < 0.01, two-way ANOVA. **D-E.** Colony formation assay. Left panel: Representative photographs of the colony formation assay using MCF-7 and MCF-7R cells expressing either control siRNA or Beclin 1 siRNA in the presence of tamoxifen or not. Right panel:Quantitative analysis of colony formation. **F-G.** Apoptosis was determined by flow cytometry after 24h tamoxifen treatment, using annexin V and propidium iodide in MCF-7 and MCF-7R cells transfected with control or Beclin 1 siRNA. **H-I.** Western blot for apoptosis related proteins of MCF-7 and MCF-7R cells transfected with control siRNA or Beclin 1 siRNA in the presence of tamoxifen (left). All experiments were repeated at least three times, and the quantitative results were analyzed by Image J software (right), and GAPDH was used as an endogenous control. **p<0.01. **J.** Wound healing assay. Representative image of wound healing assay of MCF-7 cells transfected with control siRNA or Beclin 1 siRNA in the presence of tamoxifen or not (left). Quantitative analysis of the wound closure distance (right). **K-L.** Transwell assay. Representative image of Transwell migration or invasion assays of MCF-7 cells with control siRNA or Beclin 1 siRNA in the presence of tamoxifen or not (left). Quantitative analysis of the migration or invasion rates. Data were shown as means ± S.D. from triplicate experiments (right). For D-L, **p<0.01, Student's t-test.

To investigate the possible influence of Beclin 1 on TAM resistance in breast cancer, we down-regulated Beclin 1 level of MCF-7 and MCF-7R cells via siRNA transient transfection (Figure 2A). Thereafter, alteration of TAM sensitivity was respectively evaluated by change of cell proliferation rate, apoptosis status together with migration and invasion capability. First, cell proliferation was examed by MTT assay and monolayer colony formation assay. Beclin 1 siRNA transfected MCF-7 cells (MCF-7/Beclin 1-siRNA) and MCF-7R cells (MCF-7R/Beclin 1-siRNA) were found to proliferate more slowly compared to control groups under TAM treatment using MTT assay (Figure 2B & 2C). According to MTT results, we chose Beclin 1 siRNA2 as optimum siRNA, and thus subsequent assay used this sequence targeting Beclin 1. According to the IC50 results above, we chose 2μM for MCF-7 and 10μM for MCF-7R as optimum TAM concentration for the following experiments which were close to their TAM IC50. Monolayer colony formation assay also evidenced that MCF-7/Beclin 1-siRNA and MCF-7R/Beclin 1-siRNA showed lower proliferation capability than control siRNA groups under TAM treatment (8.3±2.5 versus 52.7± 8.7 clones per well in MCF-7cells and 50.1 ±5.2 versus 74.7 ± 5.5 clones per well in MCF-7R cells, Figure 2D, 2E). Apoptosis as determined with double staining of Annexin V and PI by flow cytometry 24 h after TAM treatment in MCF-7/Beclin 1-siRNA and MCF-7R/Beclin 1-siRNA. Under TAM treatment, the rates of apoptosis cell rates in the control siRNA group and the Beclin 1 siRNA group of MCF-7 were 11.0 ± 3.2% and 22.1 ± 3.7%, respectively, whereas in the MCF-7R were 2.1±1.0% and 7.8±0.8% respectively (p<0.01) (Figure 2F & 2G). Effects of Beclin 1 on the expressions of bcl-2, cleaved-caspase3 and PARP by western blot were also presented in Figure 2H & 2I. Protein expression of cleaved caspase3 and cleaved PARP increased while the expression of bcl-2 decreased as Beclin 1 knockdown. Results of wound healing test showed that migration length of MCF-7/Beclin 1 siRNA cells group under TAM treatment were significantly shorter than control group 24h (3.7±3.4 versus 23.5±4.3 pixels) (P<0.01) (Figure 2J). MCF-7/Beclin 1 siRNA cells were also found to migrate and invade at a significantly lower rate than control cells under TAM treatment by *in vitro* migration and invasion assay (2.7±0.6 versus 12.3±2.1 cells per field in seeded 5 × 10^4^ cells for migration; 142.0±22.1 versus 248.0±44.7cells per field in seeded 1.5 × 10^5^ for invasion) (P<0.01) (Figure 2K & 2L). To consolidate our data, we overexpressed Beclin 1 of MCF-7 and found cell proliferation elevated under TAM treatment using MTT assay (Additional File 1, p<0.05).

### HER2 expression decreased after Beclin 1 down-regulation of breast cancer cells

**Figure 3:**
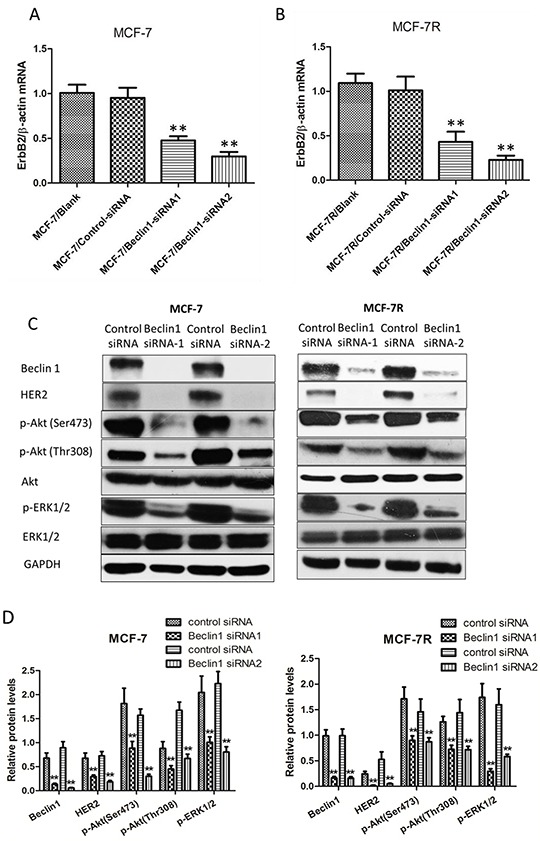
HER2 expression decreased after Beclin 1 down-regulation of breast cancer cells **A-B.** Relative mRNA level of HER2 in MCF-7 and MCF-7R cells transfected with Beclin 1 siRNA or control siRNA in the presence of tamoxifen was determined by real time qPCR. β-actin gene was used as an endogenous control for normalization. Results showed were means ± S.D. of three independent reactions. **p < 0.01, two-way ANOVA. **C-D.** Western blotting analysis of the protein levels of HER2, phosphorylated Akt, total Akt, phosphorylated ERK1/2 and total ERK1/2 in MCF-7 cells and MCF-7R cells transfected with Beclin 1 siRNA or control siRNA in the presence of tamoxifen. GAPDH was used as the loading control. All experiments were repeated at least three times. The representative results were shown in the upper panels, and the quantitative results analyzed by Image J software were shown in the lower panels. **p<0.01, Student's t-test.

TAM resistant breast cancer cells MCF-7R exhibited higher protein level of Beclin 1 along with increased HER2 than MCF-7 cells. Several studies have shown that increased expression of HER2 activates downstream pathways which can result in endocrine therapy resistance [9, 45]. To discover the potential association between Beclin 1 and HER2 expression, we examined the expression of HER2 in Beclin 1 siRNA transfected MCF-7 and MCF-7R cells treated with TAM (MCF-7/Beclin 1-siRNA and MCF-7R/Beclin 1-siRNA). Both mRNA level and protein level of HER2 were found to be decreased in MCF-7/Beclin 1-siRNA and MCF-7R/Beclin 1-siRNA cells treated with TAM (Figure 3A-3D). Also, western blot results showed downregulation of HER2 downstream effectors such as pAKT and pERK1/2 in MCF-7/Beclin 1-siRNA and MCF-7R/Beclin 1-siRNA cells treated with TAM (Figure 3C & 3D). These results suggested that the interaction between Beclin 1 and HER2 may play a crucial role in the process of TAM resistance of breast cancer cells.

### Overexpression of HER2 in Beclin 1 knockdown breast cancer cells showed lower TAM sensitivity

**Figure 4:**
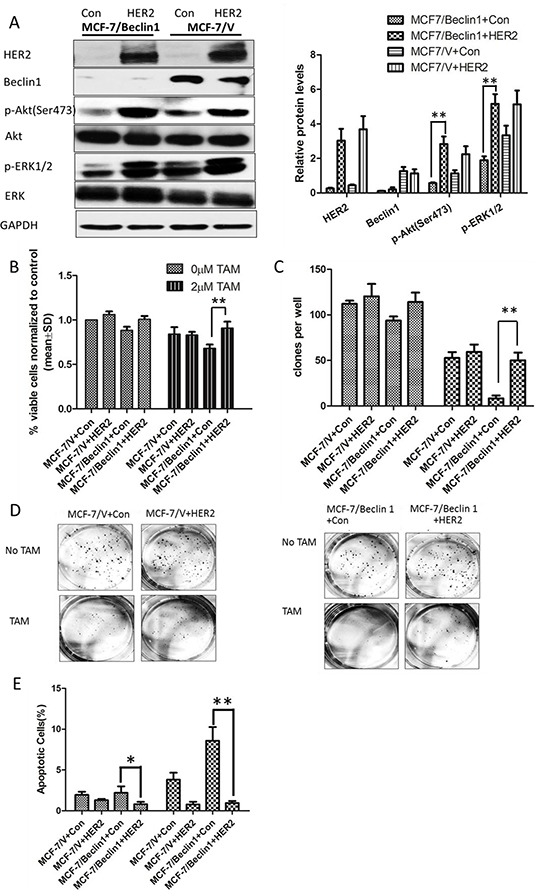

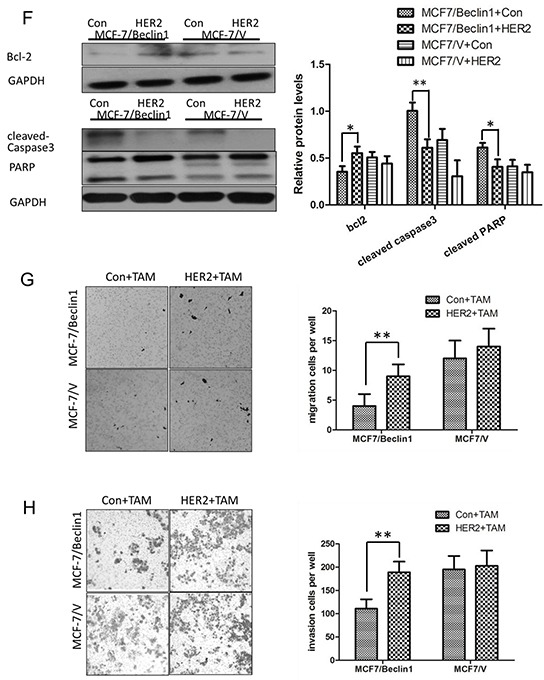
Overexpression of HER2 in Beclin 1 knockdown breast cancer cells showed lower TAM sensitivity **A.** Western blotting analysis of the protein levels of Beclin 1, HER2, phosphorylated Akt, total Akt, phosphorylated ERK1/2 and total ERK1/2 in MCF-7/V cells and MCF-7/Beclin 1 cells overexpressed HER2 or not, in the presence of tamoxifen. GAPDH was used as the loading control. The representative results were shown in the left panel, and the quantitative results analyzed by Image J software were shown in the right panel. **p<0.01, one-way ANOVA. **B.** MTT assay of proliferation in MCF-7/V cells and MCF-7/Beclin 1 cells overexpressed HER2 or not in the presence of tamoxifen or not. **p < 0.01, two-way ANOVA. **C.** Quantitative analysis of colony formation. **p < 0.01, two-way ANOVA. **D.** Representative photographs of the colony formation assay in MCF-7/V cells and MCF-7/Beclin 1cells overexpressed HER2 or not in the presence of tamoxifen or not. **E.** Apoptosis was determined by flow cytometry after 24 h tamoxifen treatment, using annexin V and propidium iodide in MCF-7/V cells and MCF-7/Beclin 1 cells overexpressed HER2 or not. **p < 0.01, two-way ANOVA. **F.** Western blot for apoptosis related proteins of MCF-7/V cells and MCF-7/Beclin 1 cells overexpressed HER2 or not in the presence of tamoxifen. The representative results were shown in the left panel, and the quantitative results analyzed by Image J software were shown in the right panel. *p<0.05,**p<0.01, one-way ANOVA. **G-H.** Transwell assay. Representative image of Transwell migration or invasion assays of MCF-7/V cells and MCF-7/Beclin 1 cells overexpressed HER2 or not in the presence of tamoxifen (left). Quantitative analysis of the migration or invasion rates. Data were shown as means ± S.D. from triplicate experiments (right). **p<0.01, one-way ANOVA.

As HER2 might be a key factor mediating Beclin 1 knockdown related TAM sensitivity activation, we overexpressed HER2 level in Beclin 1 shRNA stable transfected cells MCF-7/Beclin 1 cells via transfection of an expression vector (pcDNA3.1-HER2). Western blotting analysis confirmed the significantly increased expression of HER2 in cells transfected with pcDNA3.1-HER2, while the Beclin 1 expression was not changed (Figure 4A). Furthermore, in the presence of tamoxifen, inhibited pAkt and pERK1/2 protein of Beclin 1 knockdown breast cancer cells were reactivated after HER2 overexpression in our western blot results (Figure 4A). Then, TAM sensitivity of breast cancer cells were evaluated by change of cell proliferation rate, apoptotic status together with migration and invasion capability. Initially, cell viability study using MTT assay revealed that MCF-7/Beclin 1 cells treated with HER2 expression vector proliferated much more rapidly than control cells under TAM treatment (90.7±7.4% viable cells in HER2 expression vector group versus 68.1±4.4% in control expression vector group, p<0.01) (Figure 4B). Further we discovered that overexpressed HER2 increased colony forming efficiency of MCF-7/Beclin 1 cells in monolayer colony formation assay after TAM treatment (50.1±8.5% in HER2 expression vector group versus 8.3±3.1% in control expression vector group, p<0.01) (Figure 4C & 4D). In addition, apoptosis status of HER2 upregulated MCF-7/Beclin 1 cells were examed by Annexin V staining using flowcytometry. The rates of apoptosis cell in MCF-7/Beclin 1 cells treated with control vector group are 8.6 ± 1.7% whereas in the MCF-7/Beclin 1 cells treated with HER2 expression vector group are 1.0 ± 0.2%(p<0.01, Figure 4E). Apoptosis regulating protein Bcl-2, cleaved-caspase3, PARP were assessed by western blot, and their expression in MCF-7/Beclin 1 cells were restored after overexpressed HER2 in the presence of tamoxifen (Figure 4F). HER2 upregulated MCF-7/Beclin 1 cells were also found to migrate and invade at a higher rate than control MCF-7/Beclin 1 cells under TAM treatment by *in vitro* migration and invasion assay (8.1±2.2 versus 3.9±2.1 cells per field in seeded 5 × 10^4^ cells for migration; 189.2±23.7 versus 111.0±20.4cells per field in seeded 1.5 × 10^5^ for invasion) (p=0.07 for migration and p<0.05 for invasion) (Figure 4G & 4H).

### Beclin 1 expression was a independent prognostic predictor for ER-positive breast cancer patients treated with TAM and was positively correlated with HER2 expression

**Figure 5:**
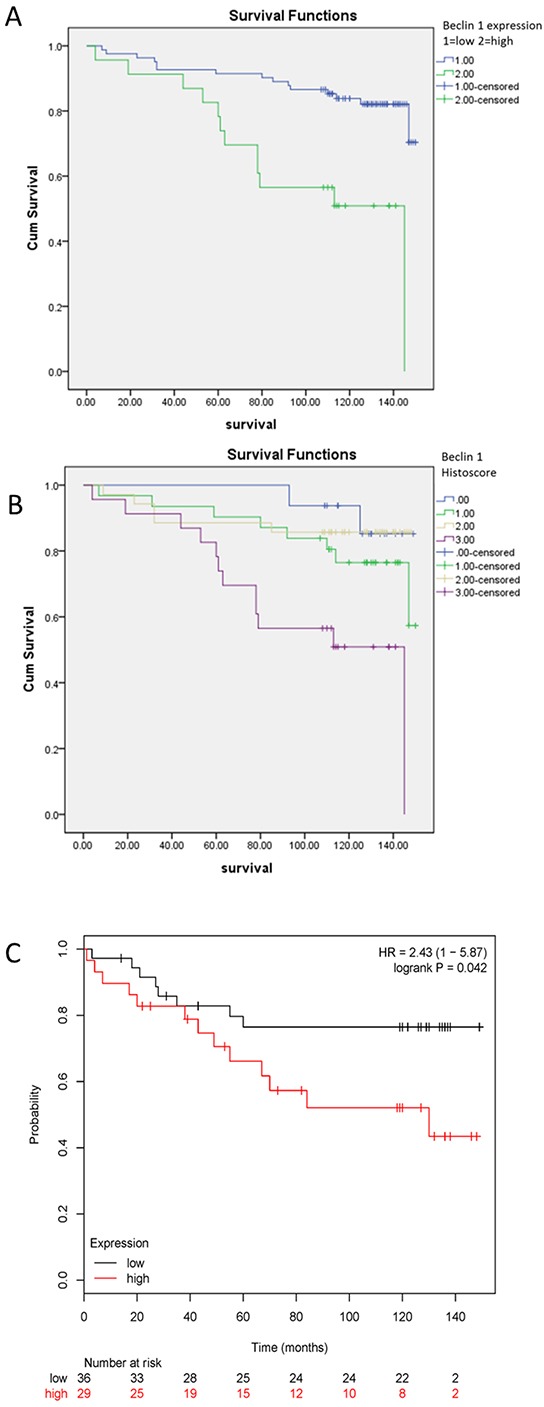
Kaplan-Meier survival analysis of Beclin 1 expression in patients with ER-positive breast cancer **A-B.** Survival analysis of Beclin 1 expression in ER-positive breast cancer patients by TMA analysis according to Beclin 1 low-or-high expression level (A) or Beclin 1 histoscore (B). **C.** Survival analysis of Beclin 1 expression in ER-positive breast cancer patients with tamoxifen treatment by Kaplan Meier Plotter database.

To verify our discovery regarding role of Beclin 1 in ER-positive breast cancer cells, we investigated relationship between Beclin 1 and patients’ survival using IHC staining of Beclin 1 of patients’ cancer tissue. To generate a rational Beclin 1 cutoff point in relation to patient outcome, the semi-quantitative IHC scores of each patient in TMA cohort were subjected to ROC analysis. Univarite analysis using Kaplan–Meier analysis evaluated significant impact of clinicopathologic prognostic variables, such as age (P=0.146), histological grade (P <0.001), tumor size (P <0.05), lymph node metastasis (P=0.476), AJCC stage (P=0.009), HER2 expression (p=0.077) on patients’survival (Table 1). Assessment of patients survival of TMA revealed that increased expression of Beclin 1 was closely associated with poor overall survival in ER-positive breast cancer patients (p<0.001 for Beclin 1 high-or-low expression and p<0.01 for Beclin 1 histoscore, Table 1, Figure 5A & 5B). The mean survival time for patients with tumor having low-expressed Beclin 1 was 134.7 months compared to 103.6 months for patients with tumor having high-expressed Beclin 1 (Table 1). Moreover, survival analysis was performed by Kaplan Meier Plotter database [46] with regards to Beclin 1 gene expression in subset of ER-positive breast cancer patients with TAM treatment (P<0.05, Figure 5C). We focused on the overall survival information of patients. The results demonstrated that high-expressed Beclin 1 gene was as well an adverse prognostic factor in ER-positive breast cancer having TAM treatment.

**Table 1:** Univariate analysis of different prognostic features in 105 patients with ER-positive breast cancer

Variable	Univariate Analysis		
	All Cases	Mean Survival (Months)	P Value
Age at surgery (years)			0.146
<=53#	60	132.6	
>53	45	122.1	
Histological grade			<0.001
G1	23	137.2	
G2	74	130.2	
G3	8	74.1	
Tumor size			0.042
<=5cm	99	130.6	
>5cm	6	81.2	
Lymph node metastasis			0.476
negative	40	132.3	
Positive	62	122.9	
AJCC			0.009
1	8	132.6	
2	62	136.5	
3	32	107.6	
4	0	—	
HER2 expression			0.077
Negative	86	131.4	
Positive	19	107.0	
Beclin 1 expression			<0.001
Low(0,1,2)	82	134.7	
High(3)	23	103.6	
Beclin 1 expression			0.002
0	16	143.5	
1	31	131.1	
2	35	132.0	
3	23	103.6	

Further multivariate Cox proportional hazard regression analysis was employed to identify the independent value of each variable for predicting patients overall survival (Table 2). Expression of Beclin 1 and clinicopathologic characteristics (including age, histological grade, tumor size, lymph node metastasis status, AJCC stage and HER2 expression) were included in multivariate analysis. As anticipated, high expression of Beclin 1 was identified as an independent risk factor of patients’ poor survival (HR=4.795, 95%CI: 1.603 to 14.341, P =0.005). With regard to other features, histological grade (P =0.001), tumor size (P =0.006) and AJCC stage (P =0.013) were shown to be independent prognostic predictors for patients overall survival (Table 2).

**Table 2:** Multivariate cox proportional-hazards analysis in 105 patients with ER-positive breast cancer

Variable	Multivariate Analysis		
	Hazard Ratio	95% confidence interval	*P* Value
Age at surgery <=53yrs(VS.>53yrs)	1.478	0.612-3.568	0.385
Histological grade			
G1	0.053	0.009-0.292	0.001
G2	0.181	0.064-0.513	0.001
G3	1	1	
Tumor size <=5cm(VS.>5cm)	7.443	1.754-31.582	0.006
Lymph node metastasis Negative (VS. Positive)	0.349	0.091-1.340	0.125
AJCC			
1	0.217	0.028-1.688	0.144
2	0.207	0.060-0.718	0.013
3	1	1	
HER2 expression Negative (VS. Positive)	0.742	0.216-2.551	0.636
Beclin 1 expression Low (VS.High)	4.795	1.603-14.341	0.005

**Figure 6:**
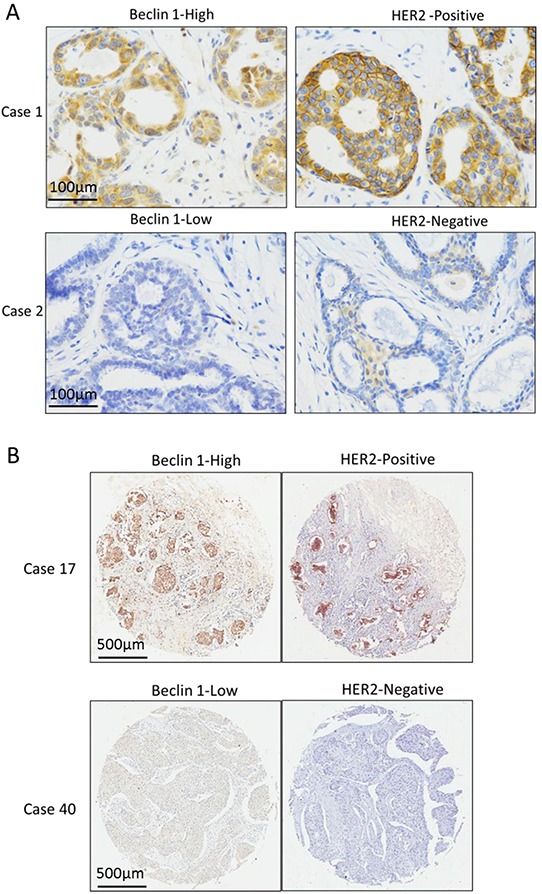
The expression relationship between Beclin 1 and HER2 in ER-positive breast cancer **A.** The Representative cases of Beclin 1 and HER2 expression in 28 ER-positive breast cancer tissue cohort. Scale bar= 100 μm. **B.** The Representative cases of Beclin 1 and HER2 expression in 105 ER-positive breast cancer TMA cohort. Scale bar= 500 μm.

Our IHC assay showed a positive correlation between protein expression levels of Beclin 1 and HER2 in ER-positive breast tissues but not ER-negative breast tissues from 41 patients including 25 ER-positive tissues and 16 ER-negative tissues (p<0.01, Table 3, Figure 6A). To further validated this result in TMA tissues, correlation analysis was done and showed a significant positive correlation between Beclin 1 and HER2 expressions in 105 ER-positive breast cancer group (p<0.001, Table 4, Figure 6B). No correlation was found between Beclin 1 expression and other clinicopathologic factors.

**Table 3:** Association of beclin 1 expression with patients’ clinicopathologic features in 41 patients with breast cancer

Variable	Beclin 1 expression			
	All Cases	Low	High	*p* Value
HER2 expression in total cases(41)				0.008
Negative	26	21(80.8%)	5(19.2%)	
Positive	15	6(40.0%)	9(60.0%)	
HER2 expression in ER positive cases(25)				0.002
Negative	18	16(88.9%)	2(11.1%)	
Positive	7	1(14.3%)	6(85.7%)	
HER2 expression in ER negative cases(16)				1.000
Negative	8	5(62.5%)	3(37.5%)	
Positive	8	5(62.5%)	3(37.5%)	

**Table 4:** Association of beclin 1 expression with patients’ clinicopathologic features in 105 patients with ER-positive breast cancer

Variable	Beclin 1 expression			
	All Cases	Low	High	*p* Value*
Age at surgery (years)				0.780
<=53(mean age)	60	49(79.0%)	13(21.0%)	
>53	45	33(76.7%)	10(23.3%)	
Histological grade				0.179
G1	23	19(82.6%)	4(17.4%)	
G2	74	59(79.7%)	15(20.3)	
G3	8	4(50.0%)	4(50.0%)	
Tumor size				0.408
<=5cm	99	76(76.8%)	23(23.2%)	
>5cm	6	6(100.0%)	0(0%)	
Lymph node metastasis				0.757
negative	40	32(80.0%)	8(20.0%)	
positive	62	48(77.4%)	14(22.6%)	
AJCC				0.500
I	8	7(87.5%)	1(12.5%)	
II	62	50(80.6%)	12(19.4%)	
III	32	23(71.9%)	9(28.1%)	
IV	0	—	—	
HER2 expression				<0.001*
Negative	86	76(88.4%)	10(11.6%)	
Positive	19	6(31.6%)	13(68.4%)	

## DISCUSSION

Here we describe a novel action for Beclin 1 in ER-positive breast cancer involving its control of HER2 signaling. We demonstrate that suppression of Beclin 1 increases tamoxifen sensitivity in ER-positive breast cancer cells *in vitro*, and that lower Beclin 1 expression predicts a better prognosis in ER-positive breast cancer patients including those receiving TAM treatment. Functionally, reduction of Beclin 1 expression enhances tamoxifen sensitivity in a HER2-dependent manner. Furthermore, a positive correlation between Beclin 1 and HER2 expression is also observed in ER-positive breast cancer cells and tissues. Taken together, our data reveal that Beclin 1 plays a regulatory role in the expression of HER2 which may contribute to enhanced tamoxifen sensitivity and favorable outcome in ER-positive breast cancer.

Beclin 1 has been reported to be associated with diverse human biological processes, for example, development, tumorigenesis, immunity and aging [47, 48]. Previously Beclin 1 has been known as a haplo-insufficient tumor suppressor which might be monoallelically deleted or decreased in many cancers, such as breast cancer. Mice with Beclin 1 heterozygous disruption had higher frequency of spontaneous tumors [24, 25]. On the other hand, Ahn et al. found that malignant colorectal and gastric epithelial cells were with higher Beclin 1 protein expression than their normal mucosal epithelial cells, suggesting the tumorigenesis role of Beclin 1 in colorectal and gastric cancer [49]. Beclin 1 has also been shown to be a promising prognostic marker for a variety of cancers. Higher Beclin 1 level has been reported to be associated with better prognosis in patients with hepatocellular carcinoma [50], high-grade gliomas [51], non-Hodgkin's lymphomas [26] or gastric cancer [28]. However, other studies had opposite results that higher Beclin 1 expression indicates a worse prognosis [29, 31] A study observed that colorectal cancer patients with excessively over- or under-expression of Beclin 1 had a remarkably poorer overall survival rate than patients with either normal or limited overexpression of Beclin 1 [30]. Furthermore, many survival analysis confirmed that Beclin 1 was a good biomarker in evaluating prognosis of solid tumors after treatments [48, 50, 52–54]. In colon cancer, Beclin 1 was an adverse factor for 5-fluorouracil treatment [31, 55]. A randomized controlled study on nasopharyngeal carcinoma patients receiving radiation and chemotherapy found that higher Beclin 1 expression related with worse prognosis [32]. However, in lung cancer cells, the inhibition of Beclin 1 lead to radiation resistance, and overexpression of Beclin 1 could enhance radiation sensitivity of cancer cells [56]. Collectively, these studies showed that the role of Beclin 1 in cancer prognosis and treatment might be tissue- and context-dependent. Additional studies are required to better understand the fundamental mechanisms. When it comes to breast cancer, there are also different opinions. In contrast to previous described role of tumor suppressor, Beclin 1 was reported to be critical for breast cancer cancer stem cell maintenance and tumor development in nude mice [34]. We focused on the role of Beclin 1 in the subtype of ER-positive breast cancer especially with TAM treatment. In our study, we have observed that the protein expression of Beclin 1 level in TAM-resistant breast cancer cells is higher than parental breast cancer cells after long-term exposure to TAM. The expression of Beclin 1 was not increased in MCF7 cells after treated with tamoxifen in 48hrs (Additional File 2). As the biological reprogramming in development of acquired TAM resistance was quite complicated, the phenomenon enlightened us for more exploration. Then we find that knockdown Beclin 1 may decelerate proliferation, enhance apoptosis and weaken migration and invasion capability of ER-positive breast cancer cells which are treated with TAM. In addition to ER-positive breast cell line MCF-7, ER-positive breast cell line ZR-75-1 showed the similar results as MCF-7 (Additional File 3). We suggested that Beclin 1 downregulation may increase the sensitivity of TAM, which is consistent with others [35, 57]. Furthermore, our results from patients suggested that lower Beclin 1 expression predicted a better prognosis of ER-positive breast cancer patients especially those with TAM treatment.

Beclin 1 play various roles such as regulating autophagy initiation, autophagosomes maturation and endosomal trafficking via different protein complexes [47, 58]. The well-known functions of Beclin 1 were always contributed to its role in autophagy. Actually, autophagy through Beclin 1 in response to specific stimuli was not necessary under all circumstances [59]. Non-canonical, Beclin 1-independent autophagy pathway might be feasible when the functions of canonical autophagy proteins were compromised [60–62] (Additional File 4). For example, resveratrol-induced autophagy in human breast cancer cell was independent of Beclin 1 but dependent on ATG7 and ATG12–ATG5 [63]. Qadir et al. used different methods to inhibit autophagy related genes to increase the cytotoxicity of 4-hydroxy tamoxifen in breast cancer cells [57]. Among these methods, knockdown Beclin 1 by siRNA was the most effective one on enhancing cytotoxicity, suggesting a specific role of Beclin 1 on endorine treatment other than the rest of the autophagy-related genes. In fact, an autophagy-independent role for Beclin 1 loss was reported to contribute to the enhanced cancer stem cell maintenance and tumorigenesis that was observed in WNT1/BECN1+/−mice [34]. Many newly identified Beclin 1-interacting proteins underscores the involvement of Beclin 1 in non-autophagic cellular processes. The involvement of Beclin 1 in receptor signaling has been previously indicated by its interaction with membrane receptors or their adaptors [35, 38–40], for example, human epidermal growth factor receptors (EGFRs) [64], the δ2 glutamate receptor and Toll-like receptors. RA Rohatgi et al. found a novel action of Beclin 1 in breast cancer involving its regulation of growth factor receptor signaling. They reported that Beclin 1 could regulate PI3P lipid levels in response to growth factor stimulation to control the rate at which growth factor receptors transit through a signaling-competent early endosome compartment. Accordingly Beclin 1 might control the intensity and duration of growth factor-stimulated AKT and ERK signaling [65]. Among these membrane receptors, the interactions between EGFRs and Beclin 1 are of great interests because signals triggering via EGFRs lead to the activation of a network of signaling cascades that are involved in cell proliferation and tumorigenesis [64]. HER2 is a key member in EGFR family signaling through its heterodimerization with other family members. HER2 pathways have been shown to play an important role in tamoxifen resistance [8] and HER2 positive breast tumors are more intrinsically resistant to tamoxifen therapy [8, 66]. Our results suggested that HER2 expression is upregulated during the generation of acquired TAM resistance of MCF-7. An identified interaction on protein level between Beclin 1 and the protein tyrosine kinase receptor HER2 was reported. Upon its interaction with HER2, Beclin 1 was found to up-regulate the phosphorylation levels of HER2 and Akt. Thus Beclin 1 may contribute to the signaling and potentially, the oncogenic activity of HER2 [67]. In light of this, our study explore the relationship between Beclin 1 and HER2. Our results revealed that Beclin 1 could modulate mRNA expression of HER2. We find that both HER2 and its downstream effectors such as pAKT and pERK1/2 are decreased in Beclin 1 downregulated ER-positive breast cancer cells in the presence of TAM. In addition, our results suggested that overexpression of HER2 in Beclin 1 knockdown breast cancer cells can restore TAM resistance. On the other hand, our results showed knockdown of HER2 could not lower Beclin 1 expression which might be uni-directional (Additional File 5). We discovered that Beclin 1 could lower HER2 expression at mRNA level, logically it might be through transcription activation, mRNA stabilization or other ways. A recent research unveiled a role of Beclin 1 in maintaining persistent activities of both NF-κB and Stat3 in the oncogenesis [68]. It was showed that Stat3 binds to its response elements at the HER2(ErbB-2) promoter to upregulate HER2(ErbB-2) transcription in breast cancer, highlighting Stat3 general role as upstream regulator of HER2(ErbB-2) expression in breast cancer [69]. In a study on breast cancer radiosensitive, HER2 was found to be co-activated with basal and radiation-induced NF-κB activity in radioresistant but not radiosensitive breast cancer cell lines after long-term radiation exposure, indicating that NF-κB-mediated HER2 overexpression is involved in radiation-induced repopulation in heterogeneous tumors [70]. Thus, Beclin 1 may act indirectly by activating other transcription factors such as Stat3 or NF-κB to modulate the mRNA expression of HER2. No study regarding genetic regulation of HER2 by Beclin 1 was reported, the mechanism involved was complicated. Further study should be performed to elucidate the exact mechanisms underlying this regulation. We report for the first time that Beclin 1 level is positively correlated with HER2 expression level in ER-positive breast cancer tissues but not ER-negative ones. We also examined the protein expressions of Beclin 1 and HER2 of three ER negative cell lines, and found no positive correlation (Additional File 3A). These results indicated the specific role of Beclin 1 in ER-positive breast cancer subtype and needed further investigation.

In conclusion, our results reveal that lower Beclin 1 expression enhance tamoxifen sensitivity via HER2 down-regulation. Beclin 1 is a prognostic predictor for ER-positive breast cancer patients treated with TAM. It may be a potential new therapeutic target for TAM resistance of ER-positive breast tumors.

## MATERIALS AND METHODS

### Cell culture

Human breast cancer cell line MCF-7 (ATCC, No.HTB-22) was obtained from American Type Culture Collection (Mananas, VA, USA). These original cells were routinely cultured at 37°C in the presence of 5% CO2 in RPMI 1640 supplemented with 10% FBS. MCF-7R cells were established by culturing cell line MCF-7 in medium plus 5μM TAM for 21 days and then separating monoclones to culture in medium plus 1μM TAM over 6 months, which was maintained in consistent medium continuously.

### Transient RNA interference

The transfection of synthetic siRNAs (25nM) for *Beclin 1* and *HER2* were performed by Lipofectamine RNAiMAX (Invitrogen, Carlsbad, CA, USA) according to the protocol of manufacturer. The sense sequences of the double-stranded *Beclin 1* siRNA were 5′GGAUGAUGAGCUGAAGAGUGUUGAA3′ and 5′GGGUCUAAGACGUCCAACA3′. *HER2* siRNAs (ID: 2046) and a scrambled siRNA were purchased from GenePharma (Shanghai, China). The siRNA most effective at depressing the *Beclin 1* mRNA level was used in the following experiments.

### Construction of Beclin 1 shRNA lentiviral vector and infection into cells

According to the transient RNA interference results, one of the candidate target sequences was selected and cloned into a pGCSIL vector (GeneChem, Shanghai, China). The recombinant virus was packaged into 293T cells using a Lentivector Expression System (GeneChem, Shanghai, China). The recombinant virus was packaged by GeneChem. Nonsilencing (NS)-shRNA was also cloned into the pGCSIL vector and used as a control (GeneChem, Shanghai, China). The shRNA sequences were 5′GGGUCUAAGACGUCCAACA3′for *Beclin 1* and 5′TTCTCCGAACGTGTCACGT3′ for control.

MCF-7 cells were cultured at 5,000 cells per well in 96-well plates for infection. Twenty-four hours later, the cells were cocultured with recombinant virus carrying Beclin 1-shRNA or NS-shRNA for 10 hours. After transfection, stable cells were selected via incubating cells with 2 μg/ml puromycin for 2 weeks. Surviving single colonies were then chose and amplified. In this study, pGCSIL-Beclin 1 shRNA was infected into MCF-7 cells to obtain Beclin-1 knockdown cells MCF-7/Beclin 1. We also used pGCSIL-NS shRNA lentivirus to infect MCF-7 cells as a negative control (MCF-7/V).

### Plasmid preparation and transfection

The coding sequence of HER2/ErbB2 cDNA was successfully cloned, which was consistent with the NCBI database. On this basis, a eukaryotic expression vector of pcDNA3.1-ErbB2 was constructed and confirmed by sequencing. Transfection was performed using Lipofectamine 2000 (Invitrogen, Carlsbad, CA, USA) according to the protocol of manufacturer.

### RNA purification and quantitative reverse transcriptase-PCR

Total RNA was extracted by TRIZOL reagent according to the protocol provided by the manufacturer (Invitrogen, Carlsbad, CA, USA). RNA concentrations were quantified by NanoDrop1000 (Nanodrop, Wilmington, Del. USA). Reverse transcription reaction was performed using 2μg of total RNA with Reverse Transcription System (Promega, Madison, WI, USA). The mRNA levels of *Beclin 1* and *HER2/ErbB2* were analyzed using GoTaqqPCR Master Mix Kit (Promega, Madison, WI, USA) in ABI PRISM 7500 Sequence Detection System (Applied Biosystems, CA, USA). The real time qPCR reaction was carried out in triplicate for each sample. Theβ-actin gene was used as an endogenous control for normalization and the mRNA levels of *Beclin 1* and *HER2/ErbB2* were determined using the 2ΔΔCt method (Livak and Schmittgen, 2001). Specific primer pairs are listed in Table 5.

**Table 5:** List of all the primer sequences used

BECN 1-F	5′-GGTGTCTCTCGCAGATTCATC-3′
BECN 1-R	5′-TCAGTCTTCGGCTGAGGTTCT-3′
ErbB2-F	5′-TGCAGGGAAACCTGGAACTC-3′
ErbB2-R	5′-ACAGGGGTGGTATTGTTCAGC-3′
β-actin-F	5′-TGAGCGCGGCTACAGCTT-3′
β-actin-R	5-TCCTTAATGTCACGCACGATTT-3′

### Western blotting analysis

Cell lysates for immunoblotting were prepared by adding lysis buffer (50 mM TriseHCl (pH 7.4), 1% Nonidet P-40, 0.5% sodium deoxycholate, 150mMNaCl, 0.02% sodium azide, and 0.1% SDS) containing protease and phosphatase inhibitors (SigmaeAldrich, St. Louis, MO, USA). Appropriate protein extracts of cell lysates were fractionated by SDS-PAGE and electro-transferred to PVDF membranes (Millipore, Billerica, MA, USA). After blocked at room temperature with 5% nonfat milk in TBS-T buffer for 1 h, the membranes were incubated with primary antibodies overnight at 4°C. The next day, the membranes were washed and then incubated with suitable peroxidaseconjugated secondary antibodies for 1 h at room temperature. After washing thrice with TBS-T buffer, antibody binding was visualized using chemiluminescence detection system as described by the manufacturer (Millipore, Billerica, MA, USA). To verify equal protein loading, the blots were stripped and reprobed for peroxidase-conjugated GAPDH antibody. Molecular weights of the immunoreactive proteins were estimated based on PageRuler Prestained Protein ladder (MBI Fermentas, USA). Experiments were repeated for at least three times.

### Cell viability assay

MCF-7 or MCF-7R cell line were established in 96-well plates. After overnight incubation, the cells were exposed to different treatments for 48 hours. When measuring cell growth, 0.5 mg/mL 3-(4, 5-dimethylthiazol-2-yl)-2,5-diphenyltetrazolium bromide (MTT) (SigmaeAldrich, St. Louis, MO, USA) was added into the medium and then cells were cultured for 4 h. Afterwards, the supernatant was removed and the formazan crystals were dissolved in 200 ml dimethyl sulfoxide (DMSO) at room temperature for 15 min. Absorbance of the solution was then tested at 570 nm wavelength using an ELx800 Absorbance Microplate Reader (Biotek, Winooski, VT, USA). A standard optical density of the control cells was considered at 100% viability. Survival was evaluated by the absorbance of the treated cells normalized to the controls.

### Monolayer colony formation assay

300 cells per well were seeded into 6-well plate in triplicate, incubated in medium containing TAM or equivalent DMSO (vehicle). Every 2-3 days the medium was replaced with fresh medium containing 2 or 10μM TAM or equivalent DMSO. After 2 weeks, the colonies were fixed with 100% methanol, stained with 0.1% crystal violet and washed with phosphate buffer solution (PBS). Visible colonies (>=50 cells) were then counted for quantification.

### Apoptosis analysis by annexin V/propidium iodide staining

At various time points, control and treated cells were collected following treatment and subjected to apoptosis measurement using the annexin V/propidium iodide (PI) detection kit (BD Bioscience, San Jose, CA, USA) according to the manufacturer's protocol. A total of 10,000 cells (within whole-cell gates) per replica (3 independent experiments) were subjected to a flow cytometric analysis to evaluate the green fluorescence of annexin V and the red fluorescence of DNA-bound PI. All the data was analyzed by FlowJo software (BD Bioscience, San Jose, CA, USA).

### Wound healing assay

The cells were seeded in six-well plates (5×10^5^/well) until the cells reached 90% confluence. Then, a cross was scraped by a 10μl sterile pipette tip in the center, rinsed with PBS three times. Serum-free medium with or without TAM was replaced immediately. Cells were allowed to migrate for 24 h, and the scratches were carefully observed and photographed. The gap lengths were also calculated by software Image J. Each cell line was measured in triplicate.

### Transwell assay

Cell migration and invasion was quantified using a previously described method (Zhang J et al., 2012). Cells were pre-starved in serum-free medium for 12 h. According to the protocol provided by the manufacturer (Millipore, Billerica, MA, USA), 700 mL medium with 10% FBS was added into the wells of a 24-well plate and 8-mmpore transwell inserts were plated into those wells for 1 h rehydration at 37°C. For invasion assay, the membranes of the inserts were coated with Matrigel (BD Bioscience, San Jose, CA, USA) at 37°C for 30 min before the rehydration. Then, starved cells were harvested with serum-free medium and cells (5 × 10^4^ for migration and 1.5 × 10^5^ for invasion assay) were seeded into the prepared inserts. After 24 h incubation at 37°C with 5%CO_2_, cells remaining inside of the inserts were removed by a cotton swab. Membranes were then fixed with 95% ethanol, stained with 0.1% crystal violet, washed with PBS. After that, they were cut from the inserts and fixed onto glass microscope slides using 50% glycerol with cover glasses. For quantification, the membranes were viewed at ×200 magnifications under light microscope. Five separate fields per membrane were selected and the number of stained cells was counted in each field.

### Tissue samples

Tissue microarray was purchased from Shanghai Outdo Biotech (Shanghai, China), which contains 105 ER-positive breast cancer specimens. Follow-up data about 9-12.5 years of these 105 cases were used for survival analysis. A total of 41 paraffin-embedded breast cancer specimens were obtained from patients undergoing curative surgery between June 2009 and May 2010 at the Cancer Center of the First Affiliated Hospital, College of Medicine, Zhejiang University, Hangzhou, China. All samples were evaluated and subjected to histological diagnosis by expert pathologists. The present study was conducted according to the Declaration of Helsinki and all procedures involving human subjects were approved by the Medical Ethics Committee in the First Affiliated Hospital, College of Medicine, Zhejiang University. Verbal informed consent was obtained from all subjects, witnessed, and formally recorded.

### IHC staining and scoring analyses

Briefly, sections were dewaxed, hydrated, and washed. After neutralization of endogenous peroxidase and microwave antigen retrieval, slides were preincubated with blocking serum and then were incubated overnight with each antibody, including rabbit anti-Beclin 1 antibody (Abcam, Cambridge, UK) and rabbit anti-HER2 antibody (Abcam, Cambridge, UK) that were diluted at 1:100. Subsequently, the sections were serially rinsed, incubated with second antibodies, and treated with HRP-conjugated streptavidin. Reaction products were visualized with 3, 3-diaminobenzidine tetrahydrochloride and counterstained with hematoxylin. Brown cytoplasmic staining for Beclin 1 and brown membranous staining for HER2 were considered to be positive immunoreactions. Beclin 1 expression level was evaluated by integrating the percentage of positive tumor cells and the intensity of positive staining. The intensity of staining was scored as follows: negative (score 0), bordering (score 1), weak (score 2), moderate (score 3), and strong (score 4). We scored the staining extent according to the percentage of positive stained cells in the field: 0–100%. The product of the intensity and extent score was considered as the overall IHC score. Immunohistochemical staining level was assessed and scored by two independent pathologists, who were blind to the clinicopathological and followup information. HER2 staining was analysed according to the American Society of Clinical Oncology (ASCO)/College of American Pathologists (CAP) guidelines, with the following categories: 0, no immunostaining; 1+, weak, incomplete membranous staining in <10% of tumor cells; 2+, complete membranous staining, either uniform or weak, in at least 10% of tumor cells; and 3+, uniform intense membranous staining in at least 30% of tumor cells. HER2 cases with strong (3+) membranous staining were considered to be positive, whereas cases graded 0 to 1+ were considered to be negative. The cases graded 2+ were further determined by initial clinical pathological FISH records. High or low Beclin 1 expression in TMA cohort was defined according to the cutoff point generated by receiver operating characteristic (ROC) analysis. High or low Beclin 1 expression in 41 breast tissue cohort was defined according to the median.

### Kaplan-Meier plotter analysis

The prognostic value of Beclin 1 in ER-positive breast cancer receiving TAM treatment was analyzed using Kaplan-Meier Plotter (http://kmplot.com/analysis/) [46]. To date, Kaplan-Meier Plotter contains information on 22,277 genes and their effect on survival in 4,142 breast cancer patients from Gene Expression Omnibus (GEO). Our study mainly analyzed overall survival patient information. Two groups with higher and lower Beclin 1 expression were compared using a Kaplan-Meier survival plot.

### Statistical analysis

Results were expressed as means ± SDs. All data were analyzed using SPSS software version 18.0. Experiments were statistically analyzed by Student's *t*-test, one-way ANOVA or two-way ANOVA. *P*<0.05 was deemed to be statistically significant.

## SUPPLEMENTARY MATERIALS FIGURES


