# Supplementary material for: Lower Beclin 1 downregulates HER2 expression to enhance tamoxifen sensitivity and predicts a favorable outcome for ER positive breast cancer
Source: Oncotarget. 2016 Aug 4;8(32):52156–77. doi: 10.18632/oncotarget.11044 (PMC5581020; doi:10.18632/oncotarget.11044)
Supplement: Supplementary file 1 [file oncotarget-08-52156-s001.pdf]

Lower Beclin 1 downregulates HER2 expression to enhance tamoxifen sensitivity and predicts a favorable outcome for ER positive breast cancer

ADDITIONAL FILES

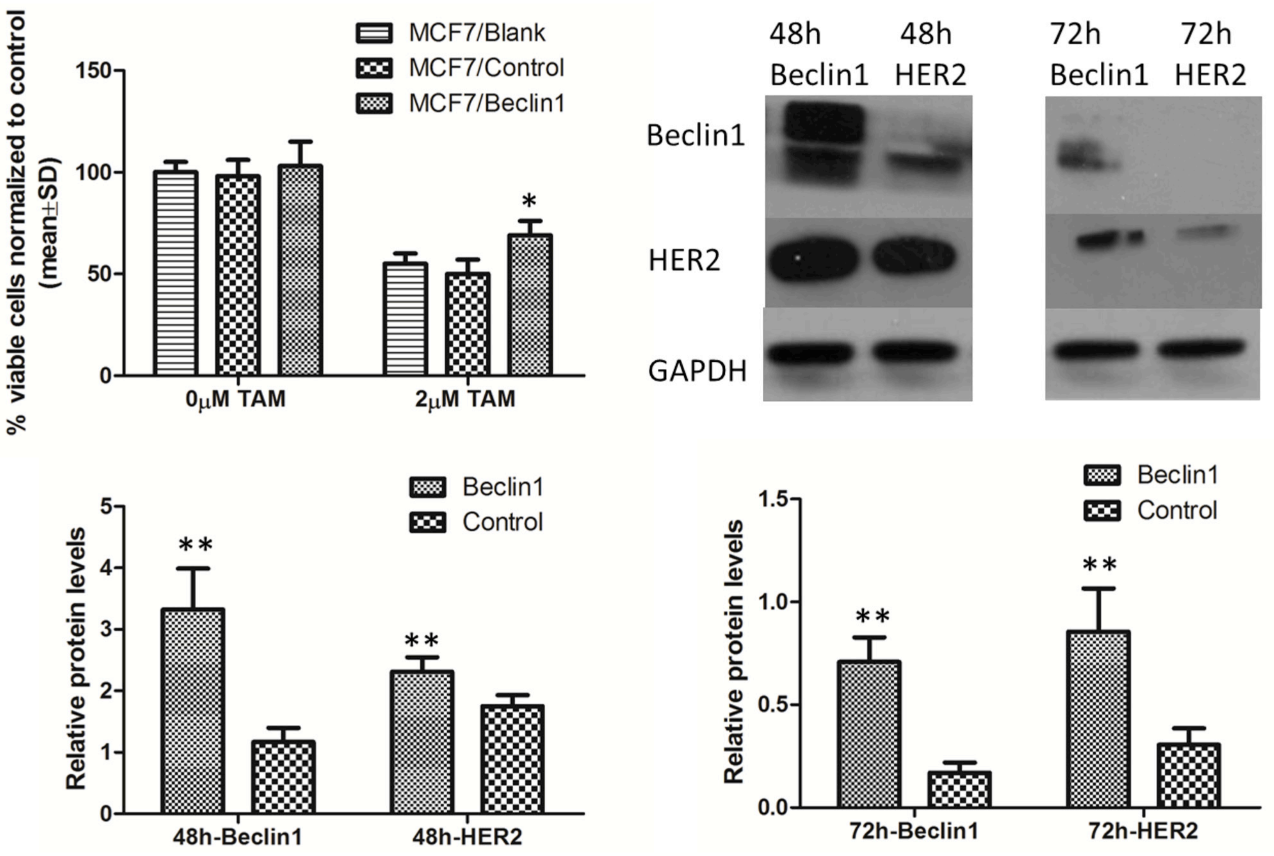

Additional File 1: Beclin 1 over-expression led to increased HER2 expression and enhanced cell proliferation of MCF-7 under TAM treatment.

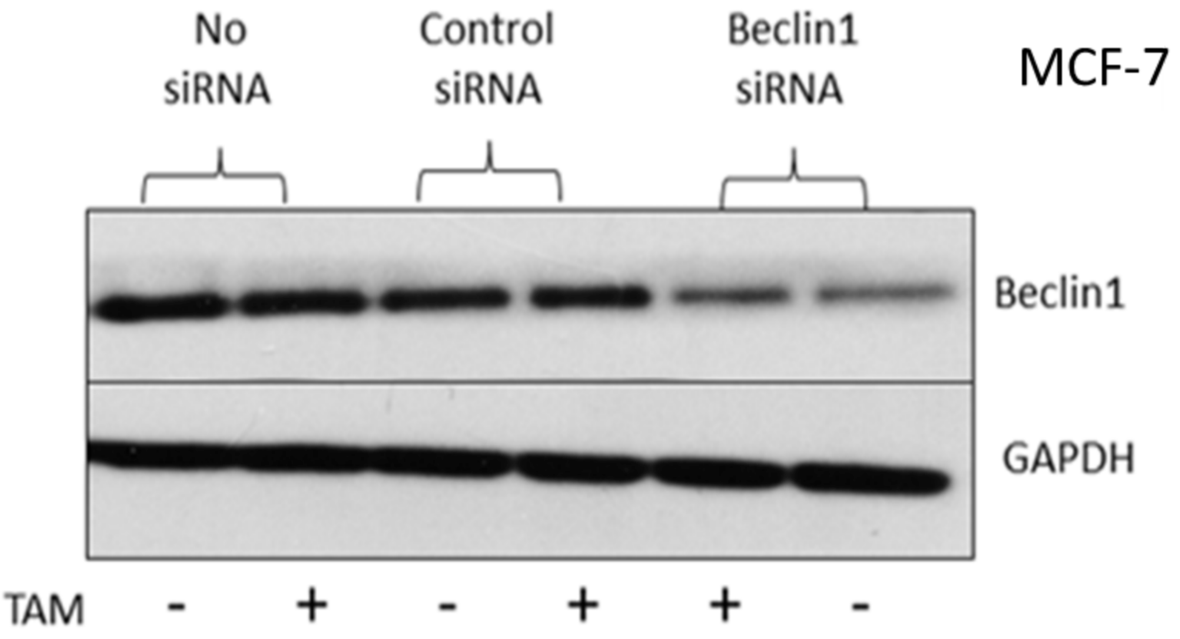

Additional File 2: The expression of Beclin1 in breast cancer cell MCF7 after treated with TAM for 48hrs.

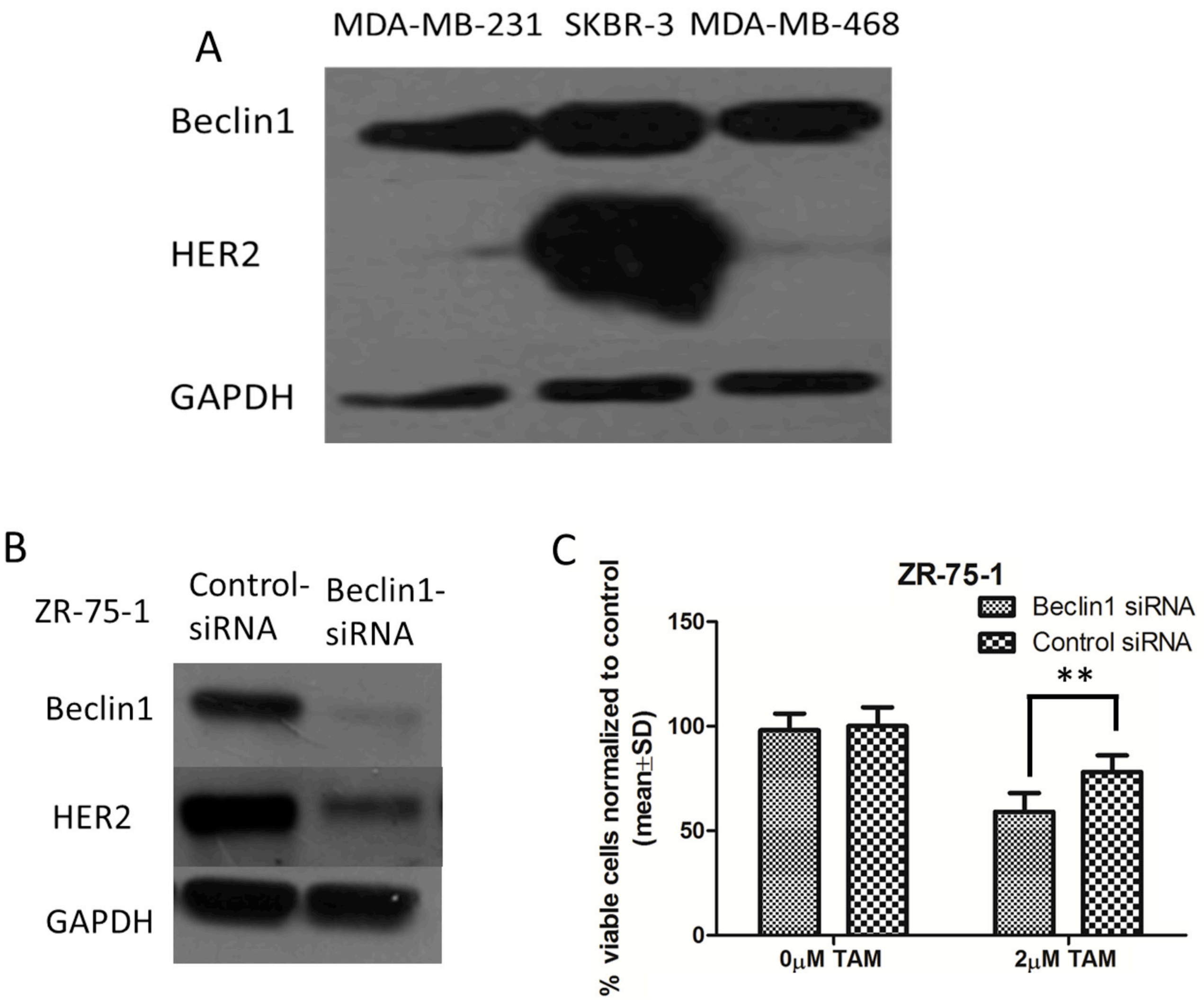

Additional File 3: Role of Beclin 1 in different breast cancer cell lines.

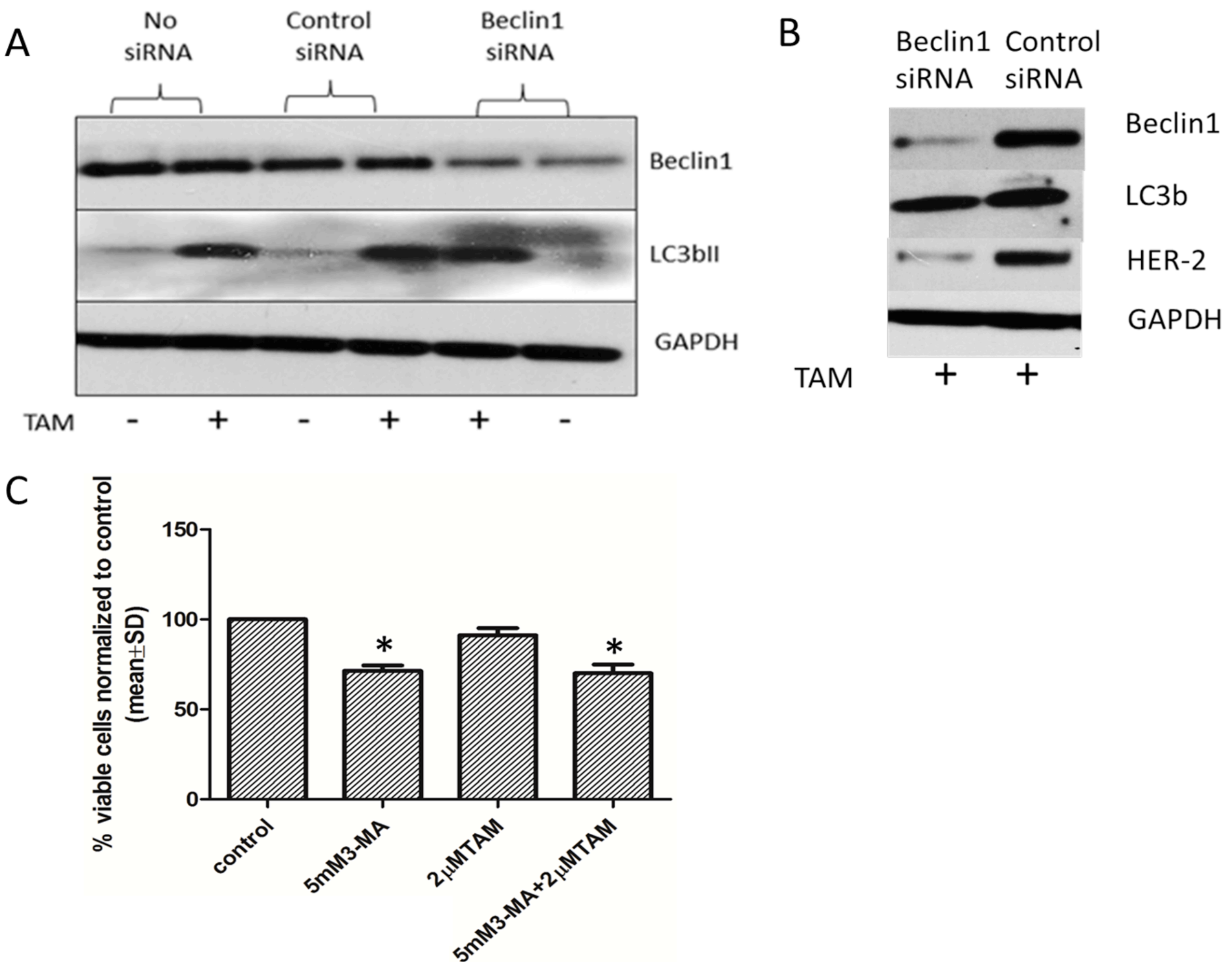

Additional File 4: Role of Beclin1 in TAM induced autophagy of MCF-7.

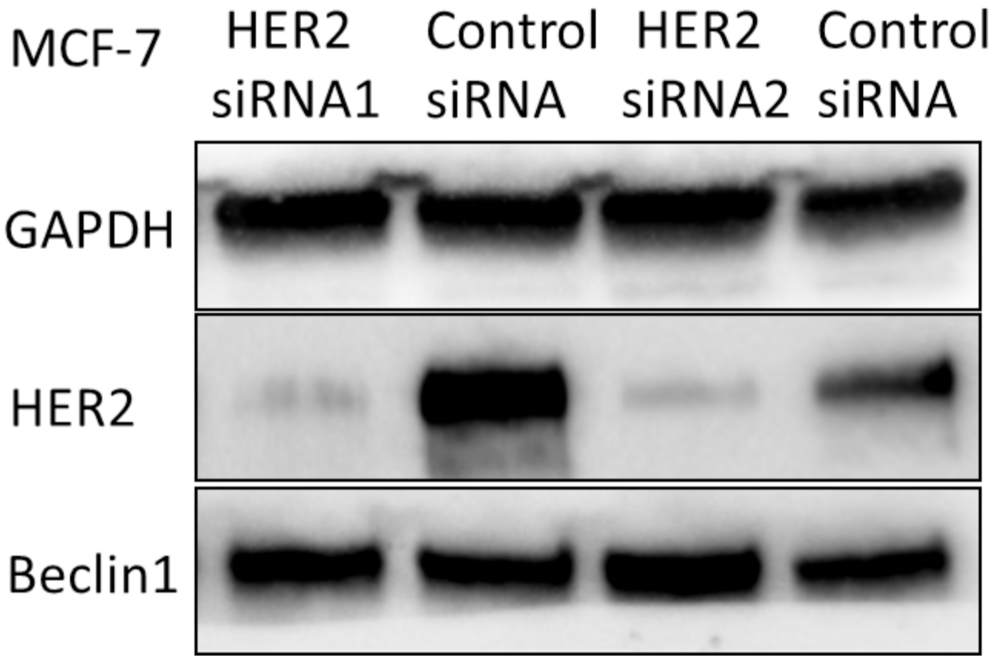

Additional File 5: The expression of Beclin 1 in breast cancer cell MCF-7 after HER2 down-regulation.
